# Towards understanding the relation between citations and research quality in software engineering studies

**DOI:** 10.1007/s11192-018-2907-3

**Published:** 2018-09-22

**Authors:** Jefferson Seide Molléri, Kai Petersen, Emilia Mendes

**Affiliations:** BTH - Blekinge Tekniska Högskola, Karlskrona, Sweden

**Keywords:** Empirical software engineering, Research practice, Reporting of research, Scientific impact, Exploratory study, Conditional inference tree

## Abstract

The importance of achieving high quality in research practice has been highlighted in different disciplines. At the same time, citations are utilized to measure the impact of academic researchers and institutions. One open question is whether the quality in the reporting of research is related to scientific impact, which would be desired. In this exploratory study we aim to: (1) Investigate how consistently a scoring rubric for rigor and relevance has been used to assess research quality of software engineering studies; (2) Explore the relationship between rigor, relevance and citation count. Through backward snowball sampling we identified 718 primary studies assessed through the scoring rubric. We utilized cluster analysis and conditional inference tree to explore the relationship between quality in the reporting of research (represented by rigor and relevance) and scientiometrics (represented by normalized citations). The results show that only rigor is related to studies’ normalized citations. Besides that, confounding factors are likely to influence the number of citations. The results also suggest that the scoring rubric is not applied the same way by all studies, and one of the likely reasons is because it was found to be too abstract and in need to be further refined. Our findings could be used as a basis to further understand the relation between the quality in the reporting of research and scientific impact, and foster new discussions on how to fairly acknowledge studies for performing well with respect to the emphasized research quality. Furthermore, we highlighted the need to further improve the scoring rubric.

## Introduction

In an ideal scientific world, we expect research reports (e.g., academic papers) to be of high quality, to present a significant contribution to the body of knowledge, and also to have their impact reflected in the number of citations (Aksnes 2006). Such scientific reports should provide the research community with new insights, describe rich cases and experiences, propose new methods or evaluate those already established. Despite differences between the different scientific disciplines, this reflects the expectation on most research fields.

For this aim, quality in the reporting of research has been investigated in a multidisciplinary context (see, e.g. Mårtensson et al. 2016; Benbasat and Zmud 1999), where several dimensions (such as credibility, contribution, communication, and conform) were reported as quality standards for scientific research. These standards reflect recommendations and best practices provided in guidelines and supporting literature; they are also the basis for instruments assessing reported studies, thus evaluating the potential impact to practice.

However, aspects other than the quality dimensions may have a stronger relation with impact factors, positively or negatively influencing them. For example, paper characteristics, such as number of authors, publication type and venue; and research aspects, such as discipline and industrial applicability, are candidates to present such an effect upon scientific impact (Aksnes 2003; Amin and Mabe 2003).

Therefore, we believe that it is important to understand more precisely how quality dimensions (herein rigor and relevance) have been evaluated and how they relate to one another, and to scientific impact. Such knowledge can contribute as an effective screening and selection criterion to guide researchers and practitioners in relation to which studies they should read and rely upon, and also to encourage them to be used as a yardstick to assess existing research fairly and accurately.

Given the need to understand how the quality standards we are using today relate to scientific impact, we conducted an exploratory study aimed to *explore the reporting of research with respect to rigor and relevance as well as its relation to scientific impact in software engineering.* In particular, the research presented in this paper makes the following contributions:

1. *Provides an overview on how *Ivarsson and Gorschek (2011) *scoring rubric has been* used to assess the *quality of primary studies* in systematic literature reviews (SLRs) and systematic mapping (SM) studies. Researchers may find this assessment useful to further develop and apply the rubric for assessment of primary studies. Furthermore, potential limitations of the rubric will become apparent.
2. *Identifies existing relations between scientific impact factors and the assessed research quality* in software engineering (SE) studies. Researchers may utilize the findings as a reflective tool for which studies to include in their related works. Furthermore, the findings may open a discussion and reflection on the current and desired relation between rigor, relevance and scientific impact.

The remaining of the paper is organized as follows: We summarize related concepts in “Background and related work” section, and describe the methods we used to obtain the data and to conduct our empirical analysis in “Research method” section. “Results” section presents the particular findings for each investigated objective. Later, we discuss the results in “Discussion and implications” section and point out some of the limitations of our work in “Validity threats” section. Finally, in “Conclusions” section, we conclude our study and provide suggestions for future work.

## Background and related work

To better understand the context in which this exploratory study is situated, we describe the related literature of three relevant themes: (1) background of empirical research in software engineering and its related challenges; (2) quality assessment of research, represented by rigor and relevance as dimensions; and (3) bibliometric analysis, as well as metrics and methods to evaluate scientific impact.

### Empirical research in software engineering

Software engineering (SE) is an emerging and in maturation sub-discipline of computing. It makes use of scientific and technical knowledge to the development and maintenance of software. SE research is mostly aimed at industrial practice, in particular, to supply practitioners with means to better decision making on whether or not to adopt software technologies and development methods (Wohlin et al. 2012; Kitchenham et al. 2015).

Similarly to other applied research fields, SE builds its body of knowledge upon meaningful evidence. This evidenced-based approach employs empirical research methods (such as experiments, cases studies, and surveys) to evaluate methods, techniques and tools (Wohlin et al. 2012; Kitchenham et al. 2015). Further, guidelines have also been proposed to support the research process (see, e.g. Wohlin et al. 2012; Runeson et al. 2012) and assess the resulting evidence (e.g. Höst and Runeson 2007; Ivarsson and Gorschek 2011).

However, like several other fields of science and engineering, empirical software engineering (ESE) has not yet developed a well-established method to evaluate the contribution of research practice (Shaw 2003; Mårtensson et al. 2016). While academic impact is often measured through citation counting Adler et al. (2009), the level of industrial impact lacks feasible standards. Notwithstanding, one expects high quality studies to produce stronger evidence, thus potentially more relevant to industry practice.

### Assessing research quality

Quality assessment is an important activity in research practice, as it ensures that the results of the assessed studies are meaningful, i.e. provide strong evidence (Dybå and Dingsøyr 2008). The quality in the reporting of research relates to the completeness of information needed to judge the study according to standards. The quality assessment process requires the use of an instrument (Kitchenham and Brereton 2013), and its results should support the reported findings and/or identify threats to the study’s validity.

Along these lines, rigor and relevance are two perspectives of quality that address how the reported research contributes to the body of knowledge, and its potential to transfer knowledge from research to practice (Benbasat and Zmud 1999). The two dimensions have been further investigated across several different domains, such as business, psychology, and social sciences (e.g. Barkham and Mellor-Clark 2000; Howard 2008; Syed et al. 2010).

On the one hand, rigor is usually emphasized more in academic than in industry environments, as it refers to the precision and correctness with which a study is reported regarding the research method used (Benbasat and Zmud 1999). To evaluate rigorousness, quality concepts such as internal validity, reliability and, contextuality are assessed according to the study design (Mårtensson et al. 2016; Mårtensson and Mårtensson 2007).

Relevance, on the other hand, represents the study’s potential usefulness in the target context. Relevant research is focused on address problems and on providing real value to practitioners (Benbasat and Zmud 1999). Despite this, evidence from several studies advocate a lack of industry interest on scientific research, e.g. (Fidel and Green 2004; Yitzhaki and Hammershlag 2004). One of the possible causes is the distance between academic research objectives and industry demands (Rainer et al. 2005). This gap can be abridged by conducting research in an environment that closely resembles the context in which it is intended to benefit. Quality concepts such as interesting and current idea, applicable results, and accessible presentation (Mårtensson and Mårtensson 2007; Benbasat and Zmud 1999) are related to relevance.

Although rigor and relevance are not the only quality dimensions related to research practice, their importance has been further stated in (Munir et al. 2014; Mårtensson and Mårtensson 2007; Benbasat and Zmud 1999). Ivarsson and Gorschek’s scoring rubrics (Ivarsson and Gorschek 2011) particularly assess the extent to which aspects related to rigor (as summarized in Table 1), and the potential for impacting the industry (described in detail in Table 2) are reported.

**Table 1:** Scoring rubric for evaluating rigor (Ivarsson and Gorschek 2011)

Aspect	Strong description (1)	Medium desc. (0.5)	Weak desc. (0)
Context (C)	The context is described to the degree where a reader can understand and compare it to another context. This involves description of development mode, e.g., contract driven, market driven etc., development speed, e.g., short time to market, company maturity, e.g., start-up, market leader etc.	The context in which the study is performed is mentioned or presented in brief but not described to the degree to which a reader can understand and compare it to another context	There appears to be no description of the context in which the evaluation is performed
Study design (SD)	The study design is described to the degree where a reader can understand, e.g., the variables measured, the control used, the treatments, the selection/ sampling used etc.	The study design is briefly described, e.g. “ten students did step 1, step 2 and step 3”	There appears to be no description of the design of the presented evaluation
Validity threats (V)	The validity of the evaluation is discussed in detail where threats are described and measures to limit them are detailed. This also includes presenting different types of threats to validity, e.g., conclusion, internal, external and construct	The validity of the study is mentioned but not described in detail	There appears to be no description of any threats to validity of the evaluation

**Table 2:** Scoring rubric for evaluating relevance (Ivarsson and Gorschek 2011)

Aspect	Contribute to relevance (1)	Do not contribute to relevance (0)
Users/subjects (U)	The subjects used in the evaluation are representative of the intended users of the technology, i.e., industry professionals	The subjects used in the evaluation are not representative of the envisioned users of the technology (practitioners). Subjects included on this level is:*i*) Students, *ii*) Researchers, and *iii*) Subject not mentioned
Context (C)	The evaluation is performed in a setting representative of the intended usage setting, i.e., industrial setting	The evaluation is performed in a laboratory situation or other setting not representative of a real usage situation
Scale (S)	The scale of the applications used in the evaluation is of realistic size, i.e., the applications are of industrial scale	The evaluation is performed using applications of unrealistic size. Applications considered on this level is:*i*) Down-scaled industrial, and *ii*) Toy example
Research method (RM)	The research method mentioned to be used in the evaluation is one that facilitates investigating real situations and that is relevant for practitioners. Research methods that are classified as contributing to relevance are: (i) Action research, (ii) Lessons learned, (iii) Case study, (iv) Field study, (v) Interview, and (vi) Descriptive/exploratory survey	The research method mentioned to be used in the evaluation does not lend itself to investigate real situations. Research methods classified as not contributing to relevance are: (i) Conceptual analysis, (ii) Conceptual analysis/mathematical, (iii) Laboratory experiment (human subject), (iv) Laboratory experiment (software), (v) Other, and (vi) N/A

### Scientometrics

Scientometrics refers to the study of measurement aspects such as performance, impact, international collaboration, etc. of scientific and technological activities based, in particular, on citation analysis (Raan 1997). Citations are used to rank scientific journals, papers, research organizations and individuals as follows (Adler et al. 2009):

1. for journals, the impact factor (IF) is given by the average number of citations for a collection of articles published in preceding years (e.g., 5-year IF is the average number of citations in the past five years);
2. for individual papers, the number of citations is used as a measure of scientific impact; however, quite often the impact factor of the journals in which those papers are published ends up being the one used as an individual paper’s surrogate measure of scientific impact;
3. for individual researchers, the most commonly used research impact measure is the h-index, i.e. the largest n for which an individual has published n articles, each with at least n citations.

Citation count is important as an indication of the influence of scientific reports. Papers cited extensively often provide insights and experiences, describe new research directions, or summarize the state-of-the-art or practice in a specific field. Wohlin conducted a series of studies to analyze the most cited articles in Software Engineering journals through the years, comparing them to highlight the similarities and differences (see, e.g. Wohlin 2009a).

Similarly, other research studies investigated aspects related to scientometrics of SE publications. Two particular studies report a census of SE publications: the first characterizes the papers published in IEEE Transactions on Software Engineering journal in the period between 1980 and 2010 (Hamadicharef 2012); and the second highlights time-related trends of SE papers listed in the DBLP database^1^ with publication dates between 1971 and 2012 (João 2014). In a different scope, the particular case of Turkish SE research is investigated in Garousi (2015).

One could expect that, due to the proximity of researchers to the scientific community, measures of scientific impact (such as citation count) would be more strongly related to academic rigor than to industrial relevance. If a relationship between relevance to practice and scientific impact is not established, this could motivate the research community to investigate metrics for assessing industrial impact, i.e. how broadly academic research addresses industry demands (Garousi et al. 2016; Osterweil et al. 2008).

Although a well-established metric, citation count faces several criticisms on evaluating scientific impact. In an article called “Stop the Numbers Game”, Parnas (2007) suggests that the citation count slow down scientific progress, as it encourages researchers to publish several superficial papers rather than a few correct and relevant ones. This issue is further investigated in Garousi and Fernandes (2017) by means of a quantitative bibliometrics assessment. The results show that particular paper characteristics (such as publication venue and language) are likely to have higher impact than citation count.

Alternatives to the citation metrics have been proposed to more fairly evaluate the impact of research. Examples of those include a taxonomy to assess the citation behavior (Bornmann and Daniel 2008), and the use of citation distribution rather than a single point measure (Wohlin 2009b).

Furthermore, critics argue that the quality assessment should be emphasized over citation counting (Parnas 2007; Aksnes 2006), but so far we only have a limited understanding of how the quality in the reporting of research is related to scientific impact. Investigations on the topic are usually focused on citation analysis of published studies (Hirsch 2005; Wohlin 2009b; Wong et al. 2009; Poulding et al. 2015).

## Research method

To investigate how reporting quality relates to scientific impact, we have first identified rigor and relevance as two relevant criteria for which data can be collected. Based on the gathered data, we employed statistical and visualization mechanisms to understand their relationship. The data used herein was obtained via a systematic search and collection of SLRs and SMs, the quality assessment scores for the primary studies they included, and additional paper characteristics.

### Research questions

This study explores two objectives related to reporting quality assessment of ESE research and its relation to the scientific impact. The objectives lead us to distinct contributions, as presented in “Introduction” section. Each contribution is guided by its own research questions.

*First contribution (C1)* provides an overview of the scoring rubrics’ use. Therefore, we aim to investigate the insights of researchers using the rubric to assess the quality of reported empirical studies. Based on this, we formulated two research questions:

1. *How the scoring rubrics were applied?* This question is focused on the methodological aspects of applying the instrument.
2. *What purpose the rubrics were used as/for?* This aims to assess the reasons for scoring the included papers.

The *second contribution (C2)* explores the existing relations between impact factors and a study’s reporting quality. A statistical model was built to test the following hypothesis:

$$\hbox {H}_{0}$$:
There is no significant relation between rigor and relevance criteria and the scientific impact of studies (i.e., normalized citations per year) in ESE research.
$$\hbox {H}_{A}$$:
There is a significant relation between rigor and relevance criteria and the scientific impact of studies (i.e., normalized citations per year) in ESE research.

### Construct measures

The variables we aim to investigate are divided into two categories: the ones assessing the quality in the reporting of research, and the ones addressing its visibility (Wang et al. 2011). In our work, research quality comes from the assessed rigor and relevance criteria, while visibility is represented by the scientific impact. For each of these, we identify candidate metrics or assessment instruments, listed in Table 3.

**Table 3:** Candidate measures for the investigated criteria

Feature	Options	Consequences
Rigor and relevance	CASP Qualitative Checklist (Casp 2016)	Address the rigor, credibility, and relevance issues through ten questions. The questions are not mapped to the quality dimensions. It was developed for Evidence-Based Medicine and is broadly applied
	Dybå and Dingsøyr (2008)	Address context, rigor, credibility, and relevance criteria. There is only one relevance question addressing the value provided for research or practice
	Ivarsson and Gorschek (2011) (selected)	Address rigor and relevance through 3 and 4 questions, respectively. Results are computed in an ordinal scale
Impact	Absolute number of citations (Adler et al. 2009)	Not appropriate to compare papers with distinct ages (i.e., published in different years)
	Average number of citations (Adler et al. 2009; Garousi and Fernandes 2016) (selected)	Citations are not equally distributed over the years
	Impact factor (Adler et al. 2009)	Journal-level metric. Provides no information on specific paper

Rigor and relevance are often evaluated by a common assessment instrument. This is usually a checklist, in which the individual questions address a particular aspect or degree. The questions can be subjective and the aspects evaluated could overlap. Moreover, other quality dimensions (e.g., originality and credibility) are also addressed.

In this study, the main concerns are related to a comparable measure for both rigor and relevance. To achieve such an aim, a quantitative measurement scale for each dimension is desirable. Considering this constraint, Ivarsson and Gorschek’s rubrics Ivarsson and Gorschek (2011) were selected. The scoring rubrics assess both reporting rigor and relevance of research in SE, as summarized in Tables 1 and 2. Ivarsson and Gorschek (2011) also conducted a validation study by applying the proposed method to an SLR on requirements engineering.

Scientific impact is the product of citation analysis, and several measures have emerged to compute it. Despite criticism, counting the number of citations is the most straightforward method to measure such dimension (Adler et al. 2009). It is important to consider the progress in the citation counts over the years since paper’s publication. During this life cycle, several factors can influence its scientific impact, such as the motivations for referencing the study (Bornmann and Daniel 2008; Wang et al. 2011) and the characteristics of the scientific communication process (Wang et al. 2011). Therefore, other paper characteristics (e.g., type and venue of publication, research method used) can act as confounding factors for this metric. However, this exploratory study does not aim to explain the causes for the citations evolution.

Mature studies are more likely to achieve higher citation count, thus a normalized metric for citations is desirable to conduct comparative analysis. The average number of citations per year is a ratio measure commonly used to compare citation counts for papers between fields (Wang et al. 2011; Garousi and Fernandes 2016). It is calculated using the arithmetic mean, i.e. dividing the absolute number of citations that a paper obtained by the number of years since its publication (Garousi and Fernandes 2016).

### Study identification and data collection

**Fig. 1:**
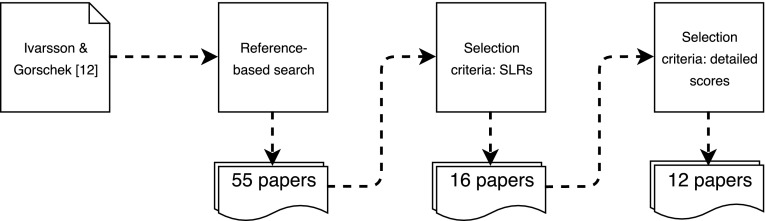
Process for identification and selection of candidate studies

First, we identified Ivarsson and Gorschek (2011) as the starting paper for a reference-based search returning 55 papers. Among those, we identified 16 secondary studies (SLRs and SMs) using the scoring rubrics to assess included primary studies. The list of candidate papers is presented in Table 4. Further, we assessed each of those candidate papers to select 12 of them providing rigor and relevance assessed scores (overall and individual scores for each scoring dimension). The process for identification and selection of the studies is illustrated in Fig. 1.

**Table 4:** Candidate papers for the exploratory study

ID	Paper type	Assessment scores	Primary studies	Data origin	Missing scores
**S1** Ali et al. (2014)	Journal (JSS)	Detailed	87	Collected from the paper	Relevance: context and research method
**S2** Barney et al. (2012)	Journal (IST)	Detailed	43	Collected from the paper	
**S3** Dogan et al. (2014)	Journal (JSS)	Detailed	58	Asked by mail	Values reported as N/A instead of 0 for Relevance: Users/Subject and Scale
**S4** Rocha and Fantinato (2013)	Journal (IST)	No	–		
**S5** Elberzhager et al. (2012)	Journal (IST)	No	–		
**S6** Galster et al. (2014)	Journal (TSE)	Detailed	196	Asked by mail	All (different methods mapped to rigor and relevance scores)
**S7** Iqbal et al. (2012)	Master thesis	No	–		
**S8** Mahdavi-Hezavehi et al. (2013)	Journal (IST)	Detailed	46	Collected from the paper	All (different methods mapped to rigor and relevance scores)
**S9** Munir et al. (2014)	Journal (IST)	Detailed	41	Collected from the paper	
**S10** Paternoster et al. (2014)	Journal (IST)	Detailed	43	Collected from the paper	
**S11** Pernstål et al. (2013)	Journal (JSS)	Detailed	38	Asked by mail	
**S12** Shashank and Darse (2011)	Master thesis	No	–		
**S13** Ullah and Ayaz (2013)	Master thesis	Overall scores only	41	Collected from the thesis	
**S14** Vakkalanka and Narayanasetty (2013)	Master thesis	Detailed	89	Collected from the thesis	
**S15** García-Mireles et al. (2013)	Journal (CLEIej)	Detailed	18	Collected from the paper	
**S16** García Mireles (2014)	Ph.D. thesis	Detailed	18	Collected from the thesis	
**Total**		**12**	**718**		

Further, we gathered the references for each of the primary studies assessed by the papers in Table 4 (totaling 718 primary studies) were stored in a dataset and is characterized by the following variables: the assessed scores for rigor and relevance (and their individual aspects), the study impact (total number of citations, year of publication, and normalized citations per year) and additional paper characteristics (type of the paper, publication venue, and length in pages). These characteristics were collected as possible confounding factors so their influence could be tested/evaluated and also discussed inside the SE domain (see, e.g. Wang et al. 2011). To enable a critical examination of our dataset and to facilitate further studies, we have made our dataset available at https://goo.gl/3y7R4l.

**Fig. 2:**
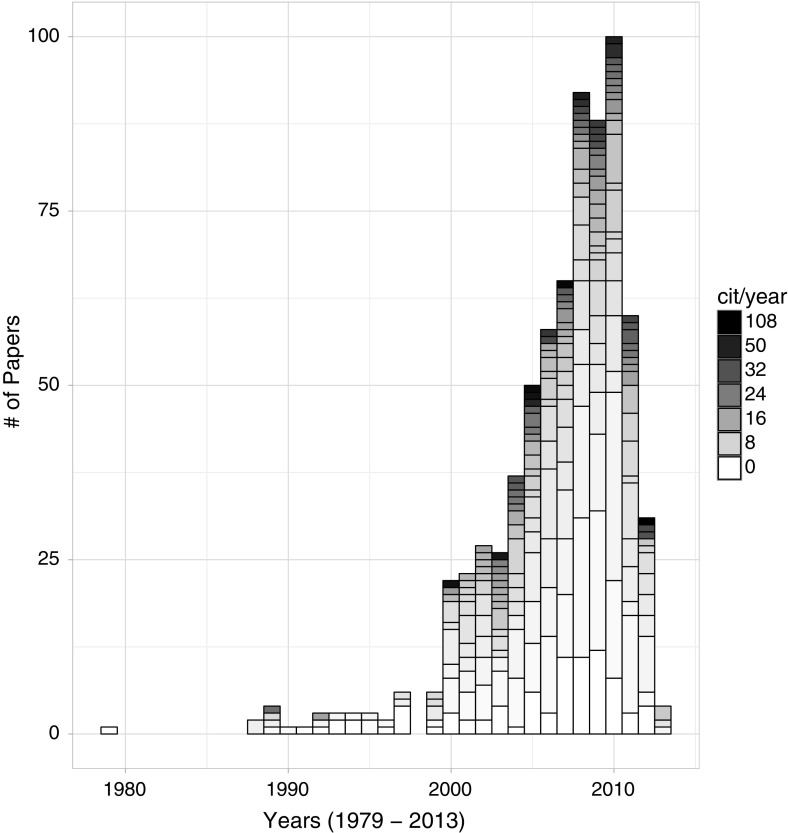
Distribution of the dataset according to the number of primary studies published in each year. The shading segments of the columns represent the normalized citation counts (cit/year), i.e. a stronger shade means a higher number of citations per year, whereas the lighter ones show the less cited papers. A legend on the right side shows a sample of the shades within the range of minimum and maximum citation counts, zero (0) and 108, respectively

As this exploratory study was carried out between mid and late 2015, we computed the normalized citation counts obtained up to 2014. This means that the most recent studies (i.e., from 2013) had at least one year of citations covered. The gathered dataset (see Fig. 2) covers both novel and mature primary studies, with citation counts varying from 0 to 108.

### Analysis

We examined the dataset and checked whether there was a significant amount of detailed scores and missing values, as summarized in Table 4. From the 16 identified studies, 7 does not provided enough information in the paper. We then contacted the authors by e-mail kindly asking them to share the list of included studies along with the scores for rigor and relevance. Three authors answered our request, thus increasing our dataset to 12 included studies. The remaining 4 studies were excluded due to the lack of detailed scores to perform the data analysis.

With regard to the *first contribution (C1)*, some of the selected studies adjusted the scoring rubrics by addressing individual aspects in a different manner:

1. Does not assess two sub-aspects related to relevance, i.e. context (C) and research method (RM). These two aspects are part of the search strategy, thus all included studies fulfill both criteria. Therefore, we replaced the missing scores with a contribution (1) score;
2. Instead of the null (0) value, the study reports some instances as not-applicable (N/A). This is mostly opposed to the rubrics’ proposed use, whereas null represents the no fulfillment of a particular rigor or relevance criterion. In this case, we replaced the N/A values for a null (0) score; and
3. Both studies map another instrument (Dybå and Dingsøyr 2008) to assess rigor, and used evidence levels as relevance scores. To collect the rigor dimension scores, we aligned each checklist item to Ivarsson and Gorschek’s (2011) rubrics. For relevance, we normalized the evidence levels (ordinal data ranging from 1 to 6) to overall relevance range (from 0 to 4). This normalization resulted in relevance scores dissimilar to those expected for the original rubrics (e.g., 0.8 instead of 1) but which are still suitable as an ordinal independent variable.

Further information regarding the application and refinement of the rubrics is given in “Overview of the scoring rubrics’ use” section, in which we discuss the first contribution of this study: an overview on how the scoring rubrics have been used to assess the reporting quality of primary studies in SLRs.

#### Relationship inference

The *second contribution (C2)* of this study is achieved by exploring the relation between rigor and relevance scores of primary studies (i.e., independent variable) and the computed citations per year for each of these primary studies (i.e., dependent variable). Based on the contribution proposed in “Introduction” section, we test the hypothesis presented in “Research questions” section. A series of statistical approaches are conducted to investigate the relation between reporting quality (i.e. rigor and relevance) and scientific impact, as follows:

*Correlation analysis:* A preliminary investigation of the relationship is made by applying correlation analysis to the dataset. The approach measures the extent of statistical covariance, i.e. which two observed variables tend to change together (Sá 2003). Spearman’s correlation coefficient (also known as Spearman’s $\rho$ or $\hbox {r}_{s}$) rates the degree of linear dependence between two variables, describing both the strength and the direction of the statistical relationship. We opted for this particular correlation coefficient because its rank approach is less subjective to particular distribution assumptions.

*Clustering variables:* A statistical approach to detect subsets of strongly correlated variables, i.e. which provide the same information, or belonging to a common group. The approach is especially useful to identify underlying structures and redundancies between variables for dimension reduction treatments (Cornish 2007). We used a hierarchical agglomerative method for data partition, iteratively aggregating the less dissimilar clusters. Later, we conducted a stability evaluation to identify which suitable clusters could be aggregated into a single dimension.

*Conditional Inference Trees (CIT)* (Hothorn et al. 2006): Finally, we use tree-structured regression models to explore the relationship between normalized citations and rigor and relevance criteria in the proposed dataset. The method is based on a unified framework for permutation tests proposed by Strasser and Weber (1999). This statistics-based approach uses non-parametric tests (Chi-squared, or ${\chi }^2$) to test the association between the candidate splitting criteria and the observed value of the dependent variable.

CITs are particularly useful to investigate ordinal variables gathered from subjective human interpretation, such as rigor and relevance scores (Hothorn et al. 2006). Besides a meaningful tool for hypothesis testing, it also provides additional features to analyze and interpret the results. The criterion for testing the hypothesis is based on multiplicity adjusted *p*-values, thus the stop criterion is maximized (e.g., with a stop criterion = 0.95 the *p*-value must be smaller than 0.05 in order to split this node). This process also ensures that the right sized tree is grown, requiring no form of pruning or cross-validation.

The CIT model was built using the *R* environment for statistical computing and the *party* package, both available from The Comprehensive R Archive Network (CRAN, http://CRAN.R-project.org/).

## Results

A total of 12 SLRs adopting the scoring rubrics to assess reporting quality of the primary studies have been selected, providing 718 instances of primary studies. Both primary and secondary studies identified were inspected to explore two particular contributions of this study.

### Overview of the scoring rubrics’ use

The first objective of this exploratory study was to review how SLRs use the scoring rubrics (Ivarsson and Gorschek 2011) to assess their included primary studies. We explore the use of the rubric focusing on two research questions (Table 5):

**Table 5:** Overview of how the Ivarsson’s and Gorschek (2011) scoring rubrics have been used

Question	Aspect	Description
RQ1. How was it applied?	As in the rubrics	Refers Ivarsson and Gorschek (2011) rubric “as is” [S10, S13, S14, S15, S16]
		Details the scoring rules [S3, S11]
		Discuss application issues [S2]
	Interpretation of the scores	Builds objective rules to assess each aspect [S1, S9]
		Maps a checklist Dybå and Dingsøyr (2008) to rigor and evidence levels to relevance scores [S6, S8]
		Two independent reviewers [S1]
RQ2. Used as/for...	Quality Assessment	Detailed assessment [S1, S3, S9, S10, S11, S13, S14, S15, S16]
		Not explicit [S2]
	Objective or Results	Research Question [S3, S9, S11, S16]
		Discussion of Results [S1, S2, S10, S13, S14]
		Implication of Findings [S6, S8]
		Study Limitation [S15]

*RQ1. How the rubrics were applied:* Most SLRs (67%) used the scoring rubrics as proposed, usually referencing the original work (Ivarsson and Gorschek 2011). Two of them (S3, S11) also presented a detailed interpretation of the scoring rules. S2 (Barney et al. 2012) discussed some issues of applying the rubrics when insufficient information regarding the study is provided in the assessed paper. Four studies used the authors’ interpretation of the scoring rubrics: (i) S1 and S9 improved the rubrics by proposing objective rules to assess the papers; (ii) S6 and S8 used another instrument for quality assessment and then mapped the outcomes to rigor and relevance scores. Finally, S1 Ali et al. (2014) discusses methodological issues on conducting the assessment activity with two independent reviewers and then using the Kappa statistic for evaluating the agreement.

*RQ2. The rubrics were used as/for:* The majority of papers (83%) used the scoring rubrics to its proposed objective - to assess the reporting quality of primary studies. Four studies (S3, S9, S11, and S16) formulated a specific research question to investigate the reporting quality of studies using rigor and relevance. Five others (S1, S2, S10, S13, and S14) discussed the resulting evidence according to the quality score of the papers. S6 and S8 also discussed the findings, however relating the assessed scores to the implications of the primary studies to research and practice. Finally, S15 discusses the quality of primary studies as a limitation of the SLR process.

It is important to highlight the application of the rubrics in studies S6 and S8, as they differ largely from the remaining papers. Both studies used a subset of Dybå and Dingsøyr (2008) checklist items to assess the quality of primary studies. We mapped the three rigor aspects to the checklist items, as presented in Table 6.

**Table 6:** Dybå and Dingsøyr’s (2008) checklist alignment to Ivarsson and Gorschek’s (2011) scoring rubrics

Rigor aspects (Ivarsson and Gorschek 2011)	Checklist items (Dybå and Dingsøyr 2008)
	Q1: Is there a rationale for why the study was undertaken?
**study context (C)**	Q2: Is there an adequate description of the context (industry, laboratory setting, products used, etc.) in which the research was carried out?
**study design (SD)**	Q3: Is there a justification and description for the research design?
	Q4: Does the study provide description and justification of the data analysis approaches?
	Q5: Is there a clear statement of findings and has sufficient data been presented to support them?
**validity threats (V)**	Q6: Did the authors critically examine their own role, potential bias and influence during the formulation of research questions and evaluation?
	Q7: Do the authors discuss the credibility and limitations of their findings explicitly?

Moreover, S6 and S8 used a 6-level scale to assess relevance according to the type of evidence provided: (1) no evidence; (2) demonstration or toy example; (3) expert opinion or observation; 4) academic study; 5) industrial study; and 6) industrial evidence. This evidence levels can be related to the relevance dimension in Ivarsson and Gorschek’s (2011) rubrics. We, therefore, normalized the evidence levels to the same scale as the relevance dimension (from 0 to 4).

### Analysis of the relationship between scientific impact and reporting quality

We conducted a progressive approach to explore the possible relationship between normalized citations and rigor and relevance scores, as follows: (1) a preliminary analysis of the correlation between dependent and independent variables; (2) the identification of underlying structures, i.e. groups of strongly correlated variables; and 3) statistical representation of the relationship based on the observed data.

**Fig. 3:**
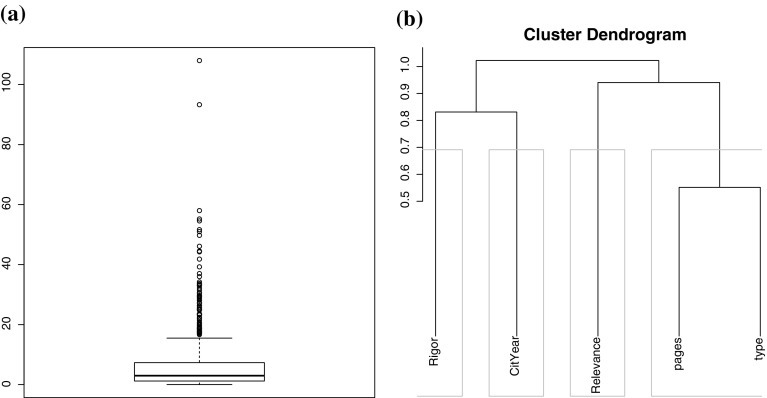
**a** Boxplot distribution of the normalized citations per year. The dots at the upper end of the plot denote the outliers, that are distant from the rest of the observations. **b** Cluster dendrogram of variables in the dataset. The gray line enclosing the variables represents suitable dimensions

A preliminary visual analysis is given by building a boxplot of the distribution of the dependent variable on the dataset, as illustrated in Fig. 3a. The majority of instances (circa 91%) are below 20 citations per year, and 64% have an impact of 5 or fewer citations per year. In addition, the boxplot presents a series of unusual highly cited papers (i.e., outliers).

Further, we conducted a correlation analysis computing Spearman’s $\rho$ coefficient to determine the strength of the relationship between the dependent and each of the independent variables separately. The observed correlation value for rigor is 0.263 denoting a weak (.2 < $\rho$ < 0.4) and positive correlation; and $-0.017$ for relevance, implying a very weak (0 < $\rho$ < 0.2) and negative correlation. Rigor and relevance also show a weak negative relation ($\rho = -0.034$).

By clustering the variables (Fig. 3b), it becomes clear that rigor, relevance and normalized citations are fairly orthogonal variables. Rigor is the variable that is more closely related to normalized citations. The paper characteristics are clustered together, suggesting that the length in pages is related to the type of publication (e.g., conferences are often subject to page limitations).

#### Hierarchical model

**Fig. 4:**
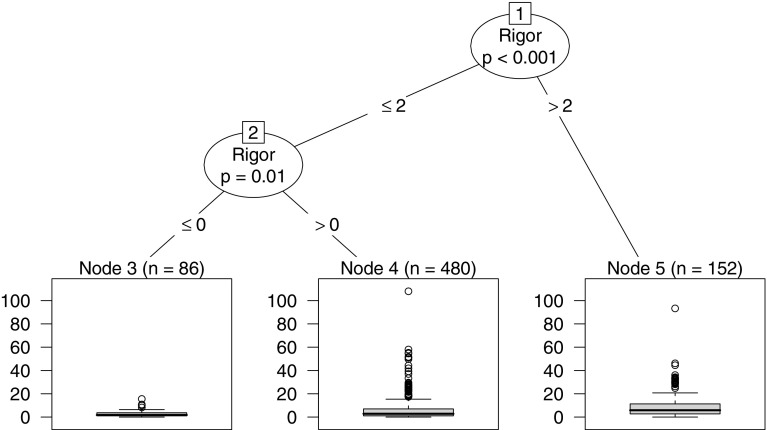
Conditional inference tree describing the relationship between rigor and relevance criteria and the normalized citations

The tree model is a hierarchical structure representing the partition of the dependent variable according to splits of the independent variables. Internal nodes are illustrated as circles, expressing the splitting criteria according to an obtained function of association with independent variables (e.g., rigor > 1.5). The terminal nodes (i.e., rectangles) represent the distribution of the dependent variable (normalized citations) according to each split. The size of the tree is automatically determined by the maximum number of internal nodes + 1 to reach the furthermost terminal node. Figure 4 illustrates the conditional inference tree model obtained from the data analysis.

The resulting tree contains two internal and three terminal nodes, including all 718 papers. The first node split the instances with a rigor score greater than 2; the remaining instances are split again by node two, on rigor greater than 0 (but still less 2). The left branch of the tree represents the data with low rigor scores (i.e., 2 or less out of 3) while the right side shows papers with higher rigor ratings. One can notice that the relevance criterion has no importance to split the dataset, as no internal node uses the variable as a splitting criterion. Table 7 details the terminal nodes, their related splitting criteria, along with characteristics of the papers contained in the node, i.e. median impact value, median paper length (in pages) and distribution of paper types.

**Table 7:** Description of the papers contained in the three terminal nodes

Nodes	Spliting criteria	Impact (cit./year)	Length (pages)	Paper type		
				Journal (%)	Conf. (%)	Others (%)
3	ri ≤ 0	1.79	9	34.8	63.9	1.3
4	0 < ri ≤ 2	2.79	10	39.3	57.0	3.7
5	ri > 2	5.83	11	44.0	52.6	3.4

The terminal nodes with higher rigor scores show a higher median number of citations per year. There is also growth on the length (in pages) and the percentage of journal papers in the nodes with higher rigor scores. On the opposite direction, conference papers are less frequent in nodes 4 and 5. Paper length and paper type characteristics are likely correlated since journal papers are often longer than conference papers.

The resulting tree model and the splitting subsets show that studies with high rigor (i.e., on the right side of the tree) often achieve greater citation impact compared to papers with lower rigor scores. Thus, the null hypotheses regarding the rigor aspect is rejected, indicating that there is a significant relation between that criterion and the dependent variable (with a *p*-value of at least 0.001). The relevance aspect, however, has no significant relationship (at $\alpha$ = 0.05) with the normalized number of citations.

#### Distribution of citation counts

**Fig. 5:**
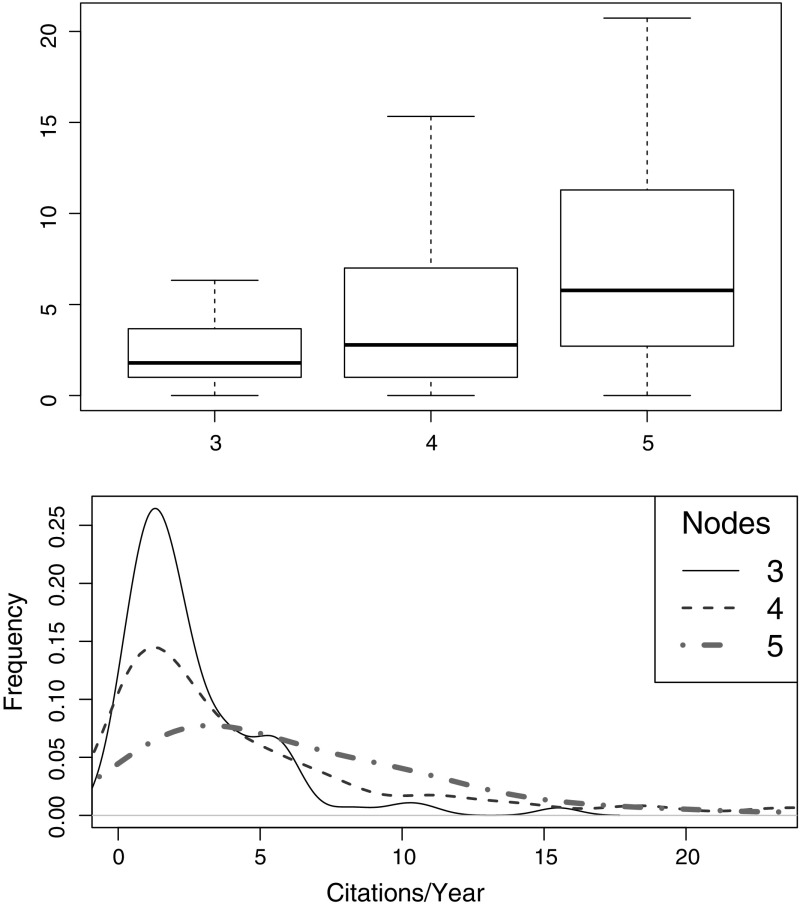
Boxplot and Density plot for the impact (i.e., normalized citations per year) according to the splitting nodes, omitting outliers

In need to better understand the distribution of the citations according to each terminal node, we refine the earlier cluster analysis, also omitting the outliers (as illustrated in Fig. 5). Looking in more detail into this helps us understand how the impact variable is distributed on each splitting subset.

The density plot shown at the bottom of Fig. 5 presents the distribution curve for each node. It is clear that node 5 has a wider distribution, assuming that papers present a higher rigor score can achieve a higher number of citations. The distribution of citations on Nodes 3 and 4 is right-skewed, thus showing that few studies on these nodes achieve a high citation count.

#### Sensitivity analysis

Further, we conducted a sensitivity analysis to test the robustness of the tree model, and increase our understanding of the relationships between the dependent and independent variables. The analysis was conducted by recalculating the outcomes under the alternative assumptions that the different studies can influence the results by introducing a significant amount of biased data (e.g., low rigor papers).

We recalculate the outcomes by removing one factor (i.e., the instances related to a particular study) at a time and comparing the produced output with the conditional inference tree presented in Fig. 4. Most of the resulting alternative trees are visually similar to our model, presenting three terminal nodes and an increase to normalized citations related to higher rigor scores. Two alternative models (i.e., removing the instances from S1 and S13) presented only one splitting node regarding rigor, still resulting in the same pattern on the normalized citations.

**Fig. 6:**
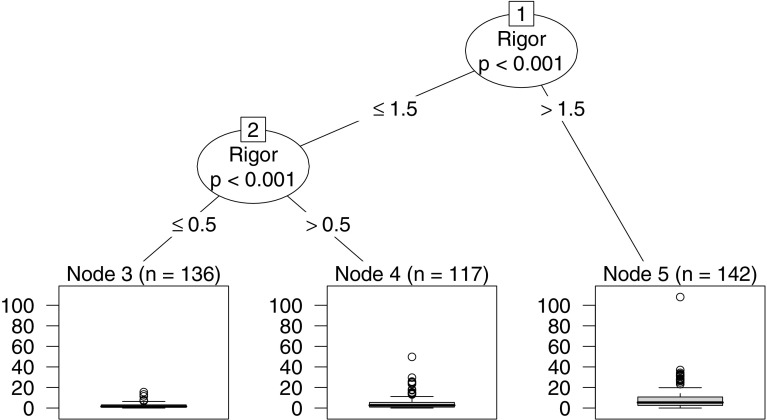
CIT excluding factors from S6, S11, and S14

Three alternative trees (i.e., removing the factors from S6, S11, and S14) resulted in models that largely differs from our original model, as shown in “Appendix”. All of them include an internal node for the relevance variable (with *p*-values of 0.034, 0.031 and 0.019, respectively), further adding splitting criteria to a subset of the rigor variable. We further grew another alternative model by removing the factors from S6, S11, and S14; to verify the impact of these instances to the model. Surprisingly, this last model is very similar to the original CIT, as shown in Fig. 6.

At large, the *p*-values and splitting criteria of the alternative models slightly differs from the original model (e.g., ri > 1.5 instead of ri > 2). The outcomes from the alternative models do not invalidate our process but raise some additional discussion on the features that could influence the results. Small divergences among the models are expected due to the differences in the number of instances, the reporting quality scores and the subjective nature of the quality assessment. Overall the sensitivity analysis shows that our model is robust.

## Discussion and implications

This exploratory study provides two distinct contributions:

C1. Identifying methodological issues of the application of the Ivarsson and Gorschek (2011) scoring rubrics.

The main purpose of the rubrics is to assess the quality of primary studies during the conduction of SLRs. However, often researchers also address the research questions and implications of the study according to rigor and relevance scores, as demonstrated in “Overview of the scoring rubrics’ use” section. Therefore, it is valuable to investigate if different quality assessment instruments (such as Casp 2016; Dybå and Dingsøyr 2008) have been used in a similar manner. This could imply a particular need to relate the assessed quality of primary studies with the evidence collected by the SLRs, as well as to propose new guidelines or update the existing ones for a comprehensive evaluation of reporting quality.

SLRs adopting the rubrics often describe the researchers’ experience and methodological issues. However no formal evaluation of the proposed instrument was done. Despite referring to the rubrics (Ivarsson and Gorschek 2011), some studies further detailed the scoring rules (S3 and S11) or even proposed some objective support (S1 and S9) to interpret the rigor and relevance criteria, thus suggesting issues related to its subjectiveness. We highlight the need for further evaluation of the scoring rubrics, investigating such issues and validating possible solutions. In particular, several researchers should score the same papers and evaluate whether they come to the same conclusions across a large set of studies. This gives confidence in the objectivity of the rubrics. Given that the rubrics are on a relatively abstract level, there may be a need to complement them with additional checklists as has been done by S1 and S9. Furthermore, S3 and S11 have seen a need to refine the rubrics. This may also affect the ability to assess the papers objectively, thus reducing biases in the assessment.

C2. Investigating the relationship between rigor and relevance.

The results of the statistical analysis show that the relation between only rigor and scientific impact is positive. Evidence obtained with the conditional inference tree model suggests a positive relation between rigor and the normalized citations per year. Despite this, we cannot infer that this relationship represents causality (i.e., increasing rigor causes an improvement on citation counting), as rigor is likely not the only criterion to impact the number of citations.

The build model also shows a lack of a significant relation between relevance and normalized citations. This result does not mean that relevance is negatively impacting the citations, but otherwise suggests that SE studies with high relevance were not particularly acknowledged by the research community with a higher number of citations. A plausible reason for this lack lies in that researchers are not aware of the relevant demands from industry (Krishnan et al. 2009; Garousi et al. 2016). Therefore, it is important to encourage researchers to conduct and to evaluate studies according to the potential impact equally on academia and practice.

Although several guides foster the conduct of realistic studies in SE (e.g. Sjøberg et al. 2007; Petersen and Wohlin 2009; Sjøberg et al. 2002), this is particularly challenging as it often requires a representative of the real usage, the involvement of practitioners, and an industrial scale. Moreover, research methods that produce results potentially more relevant usually come up with a lack of control, likely implying that achieving one criterion well may have a negative effect on another (Mårtensson et al. 2016). Our results showed some studies scoring high on both rigor and relevance, demonstrating that it is possible to ally rigor and realism.

Industrial practitioners ought to be interested in studies that have high relevance. Despite that, existing studies suggest that practitioners rarely access academic literature (Garousi et al. 2016; Rainer et al. 2005; Fidel and Green 2004; Yitzhaki and Hammershlag 2004). Consequently, to influence and value of high relevance research for practitioners, there is a need to communicate the findings to practitioners in a different way. Potential ways could be blogs, tweeting the latest research findings and linking to the sources. This would allow also measuring the impact of academic work through the feedback that could be obtained from practice through the networks.

Ultimately, the results suggest a need for better identifying and assessing the industrial impact of SE research. It is likely that this need also occurs in different applied research fields, or even across other fields of science. Cross-domain replication studies are important to test and compare our results, thus refining general findings from the particular SE-related results. For conducting such studies, proper instruments to assess the quality of studies are necessary. Ivarsson and Gorschek’s (2011) rubrics are proposed specifically for the SE domain, and its suitability to different fields have not been investigated.

A multidisciplinary approach to research quality standards is proposed and further evaluated by Mårtensson et al. (2016). This conceptual model may be the basis to identify a comprehensive set of quality criteria to assess research practice fairly. It is necessary to break its high-level concepts into finer criteria that represent actions ensuring research quality. As part of our future work, we aim to investigate the relevance of such quality criteria, in terms of its attributes, to different types of research methods employed in empirical software engineering; our goal is to support the ESE community in making informed decisions in the design of research across different SE sub-areas and groups of research.

## Validity Threats

A series of issues may influence the results of this exploratory study, such as the researchers who performed the study, the observed dataset and the measures of reporting quality and scientific impact. In the following, we consider the threats to validity, as a way to discuss the acceptance and accuracy of our findings.

*Internal validity:* The scoring rubrics were not yet evaluated by the community, though they were used to assess the reporting quality of primary studies in several SLRs and SMs. We rely on the community’s use to vouch for the acceptance of the rubrics. Given that the rubrics were not evaluated, different researchers have used them in different ways. The actual interpretation of different researchers may not be aligned. Hence, a score in one investigation may not mean exactly the same as the same score in another study. In “Overview of the scoring rubrics’ use” section we presented how the reviewers adopted the scoring rubrics when assessing studies.

Moreover, in our exploratory work, we identified three candidate studies that assessed the rigor and relevance criteria differently from proposed by the scoring rubrics. S1 considered two of the rigor aspects (i.e., context and research method) as selection criteria, as S6 and S8 adopted different assessment instruments (Dybå and Dingsøyr 2008), mapping the scores to the rigor and relevance aspects of the scoring rubrics. We also identified three relevant studies (S13, S14, and S16) originated from master and Ph.D. theses, i.e. they were not peer-reviewed. Despite this, those papers presented assessment scores and paper characteristics similar to the remaining, peer-reviewed studies. The difference in the assessment of each of these studies might have an impact on our results.

To address such potential bias, we conducted a sensitivity analysis of the statistical model (“Sensitivity analysis” section). Three alternative assumptions produced by removing one source of data (S6, S11, and S14) result in a model non-compliant with the hierarchical model. Those alternative models show extra splitting nodes related to the relevance criteria. However, most of the recalculations (9 out of 12 alternatives) produced similar results to our original tree model.

*External validity:* The data was gathered from a set of SLRs and SMs using the scoring rubrics for quality assessment of included papers, which introduces a potential bias. Though in order to (*a*) assess both rigor and relevance, and (*b*) achieve a high counter of primary studies, we based our analysis on the scoring rubrics by Ivarsson and Gorschek (2011). That is, all papers included started from the same description on how to assess rigor as well as relevance.

We do not claim that our results are representative of all software engineering literature, or to be generalizable to other fields or contexts. Though, it provided insights from a wide range of topics in SE (including economics and professional practice) across several SLRs. Also, the total number of primary studies was high (over 700).

Further studies using the rubric will get published in the future, which may influence the results. Though, with our sensitivity analysis (“Sensitivity analysis” section) we have shown that the results appear to be robust when removing or adding studies to the set. Hence, this provides confidence in the findings of our study.

*Construct validity:* Citations as a measure of scientific impact have been criticized. There are multiple purposes for citing a study, such as assumptive citations (referring to general knowledge), affirmational (confirming existing findings), contrastive (contrasting findings with existing work) and methodological (building and using method guidelines from existing work) (Bornmann and Daniel 2008). Though, the judgment of citation types is subjective and often inconsistent among reviewers (Poulding et al. 2015). In this paper, we did not make the distinction, though it may be fair to assume that an assumptive citation may not rely as much on high rigor and relevance in comparison to a methodological paper.

Beyond that, mature research papers have a head start over the novel studies as their citation counts likely increase as the years pass (Wohlin 2005). The citations evolution is not linear though, and one can argue that our scenario is unfair to the most recent papers (i.e., published in 2013). We believe that a delay of two years for the data collection does not impact the results significantly, on the contrary, is likely to hide the peculiarities of novel research (e.g., papers frequently cited shortly after their publication). Moreover, this scenario provides a representative sample that reflects the actual state of the art, in which both novel and well-established research are represented.

The scoring rubrics rely on a subjective evaluation of rigor and relevance by the reviewers. Such subjectivity could result in divergent scores depending on the reviewer’s previous experience and knowledge. In our study, we identified three duplicated primary studies assessed by different researchers in different SLRs. All of those present small divergences in the rigor or relevance scores (e.g., medium (0.5) rather than strong (1) description for the study design). One can reason that such divergences in the reviews are due to subjective interpretation of the scoring rubrics.

Moreover, rigor and relevance are not the only criteria related to the quality in the reporting of research. Additional confounding factors could be strongly related to the number of citations. Paper characteristics (i.e., length of the paper, type of publication) and research factors (i.e., research method, context, industrial applicability) showed a relation with the number of citations. We briefly addressed this validity issue in "Analysis of the relationship between scientific impact and reporting quality" section. A dendrogram demonstrated that two investigated papers characteristics (i.e., length in pages and type of publication) are related.

The impact of such and other confounding factors on the dependent variable were not further investigated, as not all of them are feasible to identify and extract. Furthermore, the rigor and relevance criteria are focused on what is reported and do not completely cover all relevant actions to be taken to, for example, evaluate a controlled experiment or case study in depth. Though, as a consequence, the rubric can be applied to diverse study types, hence including more than 700 papers, and allowing to focus on rigor and relevance at the same time.

*Conclusion validity:* During the conduct of the experiments we mostly used a single researcher to fetch the papers, collect the data, built the tree model and analyze the results. Most importantly, as the same author drew the conclusions from the gathered data, there is a risk related to the interpretation of the findings. We tried to mitigate this validation threat by discussing the preliminary results at length with the second author, and further reasoning our conclusions with the third author.

## Conclusions

In this paper, we presented an empirical study to explore the relationship between scientific impact and quality in the reporting of research. Our investigation was conducted on the findings of systematic literature reviews and mapping studies that use rigor and relevance criteria for quality assessment of primary studies, as proposed by Ivarsson and Gorschek (2011). Based on two distinct contributions, the findings of this study are:

1. We identified 16 SLRs using the scoring rubrics to assess the reporting quality or classify primary studies; wherein 12 provide detailed information on the application (i.e., the scores for each assessed study). We analyzed the selected SLRs assessing how the rubrics were applied and for which purpose. *Our findings suggest that the scoring rubric could benefit from empirical evaluation and further refinement. The use of complementary instruments also suggests that the rubrics are an early-stage quality evaluation, requiring more specific assessment rules.*
2. After that, we provided a statistical analysis of the relationship between rigor and relevance scores and the normalized citations. Evidence implies a contribution for scientific impact with increasing rigor of the studies (i.e., how the research is conducted and reported). Although we cannot elucidate this relationship at this time, the results support the raising of a question regarding the importance and worth of research showing potential impact on academia and industry, i.e. factors addressed by the relevance score. *This study provides a foundation to discuss and reflect on the current findings. For example, we may reflect on whether we should strive for a stronger relation between rigor and relevance. In case we come to the conclusion that this relation is, in fact, important, then further steps need to be taken to strengthen it. Examples could be to reflect on selecting primary studies according to the relevance criterion when conducting systematic reviews and mapping studies.*

As future work, we encourage the replication of this study across different disciplines in order to better understand the link between research quality and scientific impact. We believe that further development and evaluation of multidisciplinary instruments to assess research quality are required. Overall this line of research has the potential to lead to discussions and consensus building on how to fairly and accurately reward high quality in research practice.

## Appendix: Sensitivity analysis

Visual representations of the alternative models grown for sensitivity analysis, as detailed in “Sensitivity analysis” section:

## Footnotes

1
http://dblp.uni-trier.de
